# Editorial: Antimicrobial Resistance in Zoonotic Bacteria in Developing Countries: The Role of Food Animal Production in Public Health

**DOI:** 10.3389/fvets.2021.685281

**Published:** 2021-06-02

**Authors:** Josefina León-Félix, Gabriel Gutkind, Gabriel Arriagada, Rafael Vignoli

**Affiliations:** ^1^Laboratorio de Biología Molecular y Genómica Funcional, Centro de Investigación en Alimentación y Desarrollo, CIAD, Culicán, Mexico; ^2^Universidad de Buenos Aires, IBaViM, Buenos Aires, Argentina; ^3^Instituto de Ciencias Agroalimentarias, Animales y Ambientales – ICA3, Universidad de O'Higgins, San Fernando, Chile; ^4^Departamento de Bacteriología y Virología, Instituto de Higiene, Facultad de Medicina, Universidad de la República, Montevideo, Uruguay

**Keywords:** antimicrobial resistance, zoonotic bacteria, food animal, public health, developing countries

The control of zoonotic bacteria and combatting antimicrobial resistance are two approaches particularly relevant of the “One Health” concept (https://www.who.int/news-room/q-a-detail/one-health). Zoonotic bacteria such as *Salmonella, Escherichia coli, Campylobacter, Listeria monocytogenes, Staphylococcus aureus*, and *Brucella* are top priority for antimicrobial resistance (1).

The first group of papers in this Research Topic explores the prevalence and incidence of resistant bacteria in Latin America and the Caribbean (LAC).

Mota et al. evaluated the frequency of phenotypic antimicrobial resistance and the presence of related genes in Shiga-toxin producing *E. coli* (STEC) and *L. monocytogenes* isolated from human, food and animal sources in Uruguay. Their results indicated that 8.8% of STEC and 6% of *L. monocytogenes* were phenotypically resistant to at least one of the tested antibiotics. All phenotypically resistant *L. monocytogenes*, harbored *fosX, lin, norB, lde, mdrL*, and *fepA* resistance genes. The high load of resistance genes found, even in the susceptible isolates, indicates these two pathogens contribute significantly to the burden of antimicrobial resistance in Uruguay.

Ortega-Paredes et al. evaluated the antimicrobial resistance and prevalence of *ESBL/AmpC* and *mcr* genes from animal, food, and human components in third generation cephalosporin-resistant *E. coli* by PCR and Sanger sequencing. The high 3GC-R *E. coli* prevalence points out the risk of transmission to humans via the food chain. Implication of poultry products on the prevalence of the genes studied in 3GC-R *E. coli* should be investigated in antimicrobial resistance surveillance.

On the other hand, Mejía et al. showed, through a genomic epidemiology study that *Salmonella* Infantis ST32 is a relevant problem, with potential dissemination from poultry production farms and the food chain to the general public. They suggested Ecuadorian isolates were linked to a common ancestor, in which the participation of two pF219-like plasmids was found as responsible for distinguishing highly virulent strains.

Meanwhile, Coppola et al. conducted an investigation to detect *E. coli* isolates displaying resistance to oxyimino-cephalosporins, quinolones, and colistin in feces from livestock in Uruguay. Their results showed that the most frequently detected resistance gene recovered from animal isolates was *qnrB19*. Regarding plasmid mediated quinolone resistance genes, *qnrS1* was the second in prevalence followed by *qnrE1*, found in chickens and calves. Different β-lactamase genes were detected as responsible for oxyimino-cephalosporins resistance. This work highlights that transferable resistance genes to the three antibiotics considered critical to human health were present in feces from farm animals in Uruguay, most also reported previously in microorganisms of human origin from Uruguay.

Moreno et al. described available information to identify research and/or information gaps regarding themes of interest for antimicrobial resistance (AMR) in water in LAC. The most relevant research gaps identified are in resistance transfer, AMR surveillance, evaluating health impact of AMR, improving water treatment for AMR removal, and concluded that AMR environmental situation is driven by few countries, and therefore, research is needed in other LAC countries to better represent the region.

A second group of articles corresponded to phenotypic and genotypic characterization of resistant zoonotic bacteria.

Galarce et al. characterized antimicrobial resistance of 54 STEC isolates sampled from cattle and swine in central Chile, their findings indicate that all the isolates exhibited phenotypical resistance to cefalexin and a great proportion to colistin. The resistance genes detected were *dfrA1* and *tetA* (100%) followed by *tetB* (94.4%), *blaTEM-1* (90.7%), *aac*(6)-Ib (88.9%), *blaAmpC* (81.5%), *cat1* (61.1%), and *aac*(3)-IIa (11.1%).

Pavez-Muñoz et al. characterized antimicrobial resistance of STEC isolated from backyard production systems in central Chile, where 100% of the evaluated isolates were resistant to cephalexin and 50% to chloramphenicol, where a *stx1* type gene was present in all isolates. Several factors were identified at the farm level as responsible for determining the use of antibiotics, such as difficulties for clear disease definition and the close contact between different species. This study also constitutes the first report of resistant STEC strains circulating in the low complexity backyard production systems in Chile.

Guo et al. characterized the genome of *mcr1* positive *E. coli* isolated from pigs with post-weaning diarrhea in China by whole genome sequencing. This study found that 455 *E. coli* isolates recovered from fecal samples or small intestine contents, most were *E. coli* enterotoxigénica (ETEC), followed by atypical enteropathogenic *E. coli* (aEPEC). In the five colistin resistant isolates, three were categorized as ETEC/STEC hybrids, O3:H45, ST4214 and the remaining two as aEPEC O4:H11 ST29 and O103:H2 ST20 respectively. All displayed multiple antibiotic resistance genes, including *mcr1.1* gene and, in three cases, also *mcr3.1* presence.

Torres et al. focused their research on a strong biofilm producer strain of *S. aureus* (Sa1FB) associated with subclinical bovine mastitis in Colombia. The major differences with a reference strain were found in the number of mobile genetic elements, that could increase mutations, pathogenesis, and adaptability to new hosts, representing a risk for transmission of resistant *S. aureus* to people by milk consumption obtained from infected animals.

Umair et al. quantified antibiotics use in two large corporate dairy farms from Pakistan, reporting that the amount of antibiotic used was considerably higher than in similar studies around the world. Most used antibiotic classes were aminoglycosides, penicillin and tetracyclines, and 43% of the active principles used were critically important antimicrobials for human medicine, constituting the first study of its kind in Pakistan.

In conclusion, this eBook provides new insights about the frequency of antimicrobial resistance and other pathogenic elements of various strains of *Salmonella, E. coli* (mainly STEC), *L. monocytogenes, S. aureus*, in cattle, poultry, swine, dairy farms, and other sources, such as water samples, mainly in Latin America and the Caribbean. The evidence presented here highlights that even no coordinated studies have been sustainability undertaken. Antimicrobial resistance burden is very high, with implications not only on human health, but also potentially compromising production systems sustainably in the next future. Coordinated local, regional, and global actions regarding the use of antimicrobials in the production of food from animal origin are necessary and implementing precise diagnostic strategies would allow establishing clear and forceful guidelines that lead to the efficient use of antibiotics.

## Author Contributions

All authors listed have made a substantial, direct and intellectual contribution to the work, and approved it for publication.

## Conflict of Interest

The authors declare that the research was conducted in the absence of any commercial or financial relationships that could be construed as a potential conflict of interest.
